# Comprehensive Analysis of Insertions in BVDV and Implications for Non-Homologous Recombination Mechanisms

**DOI:** 10.3390/v18080886

**Published:** 2026-08-12

**Authors:** Fedor Perelygin, Yulia Aleshina, Ekaterina Chistiakova, Artem Orlov, Alexander Lukashev

**Affiliations:** 1 Martsinovsky Institute of Medical Parasitology, Tropical and Vector Borne Diseases, Sechenov First Moscow State Medical University, 119435 Moscow, Russia; fedor959@gmail.com (F.P.); orlov.artem.vl@gmail.com (A.O.); alexander_lukashev@hotmail.com (A.L.); 2 Faculty of Bioengineering and Bioinformatics, Lomonosov Moscow State University, 119234 Moscow, Russia; katyachistiakova.05@mail.ru

**Keywords:** bovine viral diarrhea virus, cellular mRNA insertions, non-replicative recombination, ER stress, virus emergence

## Abstract

Bovine viral diarrhea virus (BVDV) is a pathogen of globally significance in cattle that has two biotypes: non-cytopathogenic (non-cp) and cytopathogenic (cp). The cp biotype arises from the non-cp through genome rearrangements, which frequently involve the insertion of host cellular RNA sequences, often accompanied by viral genome duplications. Here, we performed a systematic analysis of insertions across all complete BVDV genomes available in GenBank. Despite a 10-fold increase in available sequences over the past 10 years (from 59 to 670), the repertoire of known rearrangements associated with the cp phenotype has expanded only modestly, with insertions occurring predominantly at five conserved genomic hotspots. Notably, independent acquisitions of similar insertions—such as *DNAJC14* (Jiv), ubiquitin-like sequences, the NS4B-NS5A cleavage site, and the PYPDPQTLG motif—in phylogenetically unrelated virus lineages reflect a limited number of permissive sites. Our analysis further demonstrates that many insertions are mosaic and suggests multiple recombination events that are poorly compatible with template switching by viral polymerase. Mechanistically, we propose that non-replicative recombination provides the most consistent explanation for the observed insertion patterns, particularly common coupling of cellular RNA acquisitions with viral sequence duplications. Both the steric proximity of virus replication to the endoplasmic reticulum and the RNA end requirements (2′-3′ cyclic phosphate and 5′-OH) suggest that non-replicative recombination is likely mediated by the endoplasmic reticulum-associated IRE1 RNase and RtcB ligase, which are responsible for the alternative cytoplasmic splicing of cellular mRNA.

## 1. Introduction

Bovine viral diarrhea virus (BVDV), a member of the genus *Orthopestivirus*, family *Flaviviridae*, is a globally prevalent pathogen that can infect various artiodactyls, primarily cattle [1,2,3,4,5,6,7]. BVDV produces two distinct infection profiles. Adult immunocompetent animals have a brief and relatively mild acute infection and recover completely, resulting in seroprevalence rates of approximately 50% in most countries [8]. However, when a pregnant cow is infected, the fetus can also be infected. If this happens before the maturation of the immune system, a persistent infection is established [9]. It manifests with limited symptoms (usually moderate growth retardation), despite active replication of the virus, and the persistently infected animal can live for many years. However, under certain conditions the virus can also cause a lethal infection. Two disease profiles in a persistently infected animal are linked to distinct biotypes of the virus. Persistent infection is caused exclusively by a non-cytopathogenic (non-cp) biotype of the virus, termed so because it does not cause cytopathic effect in a cell culture. If a persistently infected animal is superinfected with a cytopathogenic (cp) virus, it develops a severe mucosal disease, which leads to death within a few weeks [10]. A cp virus may emerge in the course of persistent infection from a non-cp virus de novo or be transmitted to an animal infected with a non-cp virus from another animal. Emergence of a cp virus is the result of rearrangements in the viral genome that promote excessive processing of viral protein NS2-3 and increase production of NS3 [11,12]. Mature NS3 binds to voltage-dependent anion channel 1 (VDAC-1) and drives Ca^2+^-dependent permeability transition, leading to Ca^2+^ overload and membrane depolarization [13]. Mitochondrial permeability transition pore (mPTP) opens, and cytochrome c-initiated caspase cascade activates the mitochondrial pathway of apoptosis. NS2-3 overprocessing is often caused by insertion of *DNAJC14* (DnaJ heat shock protein family member C14) gene fragment—encoding a cellular chaperone also termed Jiv (J-domain protein interacting with viral protein [14])—into the NS2-encoding region of the viral genome [15]. However, many alternative types of rearrangements producing the cp phenotype have been described, such as insertion of ubiquitin/ubiquitin-like domains [16], other RNA fragments, and duplication of various viral genome fragments [12]. Additionally, the rearrangements can result in variants lacking self-sufficiency due to deletion of vital regions. These genomes are not capable of sustained circulation among animals that are not persistently infected with a non-cp variant [17,18]. Cp viruses exemplify a local fitness gain—they replicate faster than non-cp viruses within an individual animal—but this represents an evolutionary dead-end [19], likely because they cannot control the interferon response [20] and thus are not capable of sustained circulation. Each *DNAJC14* insert is unique in terms of the length and boundaries of the inserted gene fragment, with the smallest known functional *DNAJC14* fragment comprising just 90 amino acids (Jiv90 in NADL strain), but only a few loci in the genome are suitable for naturally occurring insertions or other modifications [21,22]. The precise mechanisms that produce *DNAJC14* insertions and other alterations are not known.

Recombination is one of the major evolutionary mechanisms that has been described in most viruses [23,24]. The primary mechanism of homologous recombination is copy-choice, when polymerase stalls, dissociates from a template and resumes synthesis on another template [25,26].

Alternatively, virus genome fragments may be joined in the absence of virus replication by non-replicative recombination (NRR) [27]. This mechanism was originally shown in poliovirus and then confirmed in BVDV and hepatitis C virus [28,29]. NRR can yield viable viruses if a cell is transfected with overlapping genome fragments, which are joined by a cryptic cellular mechanism in an apparently random pattern. RNA fragments may be linked upon truncation of their termini, implying a concerted action of an RNase and a ligase, and often creating a duplication of virus genome fragment. These initial genomes recovered with a duplication are subsequently resolved by classical (strand-switch) homologous recombination that removes duplications and can yield an original genome without a trace of recombination [30]. NRR can also join virus ends precisely, but only if the 3′ end of the 5′ fragment contains a phosphate or a cyclic phosphate [31]. While the possibility of NRR is well proven, its actual incidence and impact on virus evolution remain unknown, because it is difficult to distinguish two mechanisms in natural settings, especially as one type of recombination is hypothetically resolved by another.

BVDV is an example of a “natural test lab”, where viruses replicate in persistently infected animals under a limited selection pressure, producing numerous genome rearrangements [32]. Some of the cp biotype variants provide a local replicative advantage to the virus, but are not fit for sustained circulation, and cannot displace other viruses globally. The structure of BVDV inserts was comprehensively reviewed from the point of view of BVDV biology [12]. Here, we revisited this topic aiming to find new insights into recombination mechanisms.

## 2. Materials and Methods

### 2.1. Data Collection

Genome sequences of pestiviruses (taxid: 11095) with lengths greater than 10,200 nt were downloaded from the GenBank database as of 25 October 2025. Artificially constructed sequences, patent records, and sequences with significant ambiguities (more than 1% of ambiguous nucleotides in RdRp, E1 and E2 coding sequences) were discarded. For GenBank entries with no annotated coding sequences (CDS), open reading frame encoding a single polyprotein was predicted using ORFfinder 0.4.3 [33]. The final dataset included 1810 pestivirus genomes with a complete or nearly complete coding sequence.

To reduce redundancy and facilitate phylogenetic analysis, sequences were clustered at an 84% identity threshold using CD-HIT v4.8.1 [34], resulting in 103 sequences. Then, a maximum likelihood (ML) phylogenetic tree for RdRp sequences was built using IQ-TREE v3.0.1 [35]. The best-fit substitution models were selected automatically under the Bayesian Information Criterion (BIC) using the ModelFinder tool [36] integrated into IQ-TREE, and branch support was assessed by an ultrafast bootstrap approximation with 1000 replicates [37]. RdRp sequence from hepatitis C virus (*Hepacivirus hominis* HK2cc, accession MG717927.1) was used as an outgroup. Pestivirus species were assigned based on phylogenetic grouping. This enabled creating a dataset for bovine viral diarrhea virus 1 and 2 (BVDV-1, BVDV-2; *Orthopestivirus bovis* and *Orthopestivirus tauri*, respectively) and Hobi-like viruses (BVDV-3, *Orthopestivirus brazilense*) comprising 566 sequences.

### 2.2. Search for Cellular Insertions and Analysis of Polyprotein Domain Architecture

For the initial screening of cellular insertions, Pfam domains were searched in the BVDV dataset (N = 566) using hmmscan program of HMMER v3.4 software [38] against the Pfam-A database with an E-value threshold of 1 × 10^−5^. Eukaryotic domains identified in the dataset were downloaded from Pfam and merged with a set of custom HMM profiles for mature viral proteins. To generate these custom HMM profiles, representative genomes of the genus *Orthopestivirus* (N = 21) according to the ICTV Virus Metadata Resource (https://ictv.global/vmr; accessed on 20 September 2024) were downloaded. Mature protein sequences were extracted from fully annotated genomes; for those genomes lacking detailed annotation, we retrieved the individual protein sequences via homology searches of annotated mature proteins against translated CDS (polyproteins) using jackhmmer (HMMER v3.4 software [39]). The obtained viral proteins were clustered using the Markov Cluster Algorithm [40,41] based on a pairwise similarity matrix derived from BLASTp v2.16.0+ bitscores (E-value ≤ 1 × 10^−3^) [42], which yielded 11 clusters corresponding to the canonical pestivirus mature proteins. For each cluster, multiple sequence alignments were generated with Clustal Omega 1.2.4 [43], and the final HMM profiles were built from these alignments using hmmbuild. The complete pipeline and profile database (also includes profiles of all *Flaviviridae* mature proteins) are available at https://github.com/orlovartem/Flaviviridae_PPHMMDB (accessed on 6 July 2026). A more detailed analysis of the polyprotein domain architecture in BVDV was then performed using hmmscan against this merged database. For visualization, the online tool DomainAnalyser v2.0 [44] was used; this tool runs hmmscan and visualizes the domains (E-value ≤ 1 × 10^−5^).

### 2.3. Representative Multiple Sequence Alignment for BVDV Dataset

Coding sequences of BVDV were translated and aligned using MAFFT v7.525 [45]. The resulting alignment was manually inspected using JalView v2.11.5.1 [46]; mature viral proteins and cellular gene insertions were annotated, and manual adjustments were made to ensure correct alignment of the mature protein regions. A codon-based alignment of the coding sequences was then generated from the amino acid alignment using pal2nal v14 [47]. The fragments of alignments with insertions were visualized in JalView.

### 2.4. Analysis of Insertion Lengths

The lengths of insertions and their locations were determined using a custom Python script. Each column of the multiple sequence alignment was processed as follows: if the fraction of gaps in a column exceeded a threshold (0.7), a counter was initiated to track the length of continuous non-gap columns. When the length of such a continuous non-gap region reached a minimum length (one amino acid), it was flagged as a putative insertion. Overlapping insertions were merged into one. For each insertion, the script calculated exact alignment coordinates and length. If domain annotations (in .hmm format) were provided, the location of insertions relative to conserved domains were also recorded. The code is available at https://github.com/WizardFPS/Systematic-analysis-of-insertions-across-pestivirus-genomes (accessed on 6 July 2026).

The lengths of insertions were visualized using matplotlib.pyplot v3.10.6 package for Python v3.13.9.

### 2.5. Phylogenetic Analysis

Sequences of E1, E2 and RdRp mature proteins were excised from the amino acid alignment of polyproteins. For each alignment, maximum likelihood (ML) trees were calculated using FastTree v2.2.0 [48] with default parameters.

To display independent cp insertions, a reduced BVDV dataset was created. Polyprotein sequences of BVDV without cp insertions (N = 511) were clustered at a 90% identity threshold using CD-HIT v4.8.1, resulting in 22 clusters. Polyproteins containing cp insertions, along with their closely related neighbors on the RdRp phylogenetic tree, were then manually added to the reduced dataset, giving a total of 78 sequences. Finally, polyprotein sequence of border disease virus was added to the resulting alignment as an outgroup. The reduced alignment was extracted from the representative polyprotein multiple sequence alignment for BVDV dataset. Then, the alignment of RdRp was extracted from the polyprotein alignment, and the ML phylogenetic tree was built using IQ-TREE v3.0.1 [35]. Default parameters were used, except for ultrafast bootstrap replicates set to 1000 and sequence type set to codon. Border disease virus (GenBank accession: MF102260) was set as an outgroup.

Phylogenetic trees were visualized using FigTree v1.4.4 [49].

### 2.6. Recombination Detection

Recombination analysis was performed on the BVDV dataset (N = 566) using RDP4 (Recombination Detection Program version 4) v101 software [50]. A full exploratory analysis was conducted with default parameters, and virus sequences were specified as linear.

To confirm recombination events involving the loss of a long insertion, separate phylogenetic trees were constructed for the recombinant sequence, its major and minor parents (as identified by RDP4), and an outgroup. A similarity plot was generated using SimPlot v3.5.1 software to calculate *p*-distances with a sliding window of 350 nt and a step size of 10 nt.

## 3. Results

### 3.1. The Repertoire of Insertions in BVDV Genomes Is High, Yet Limited

**Figure 1:**
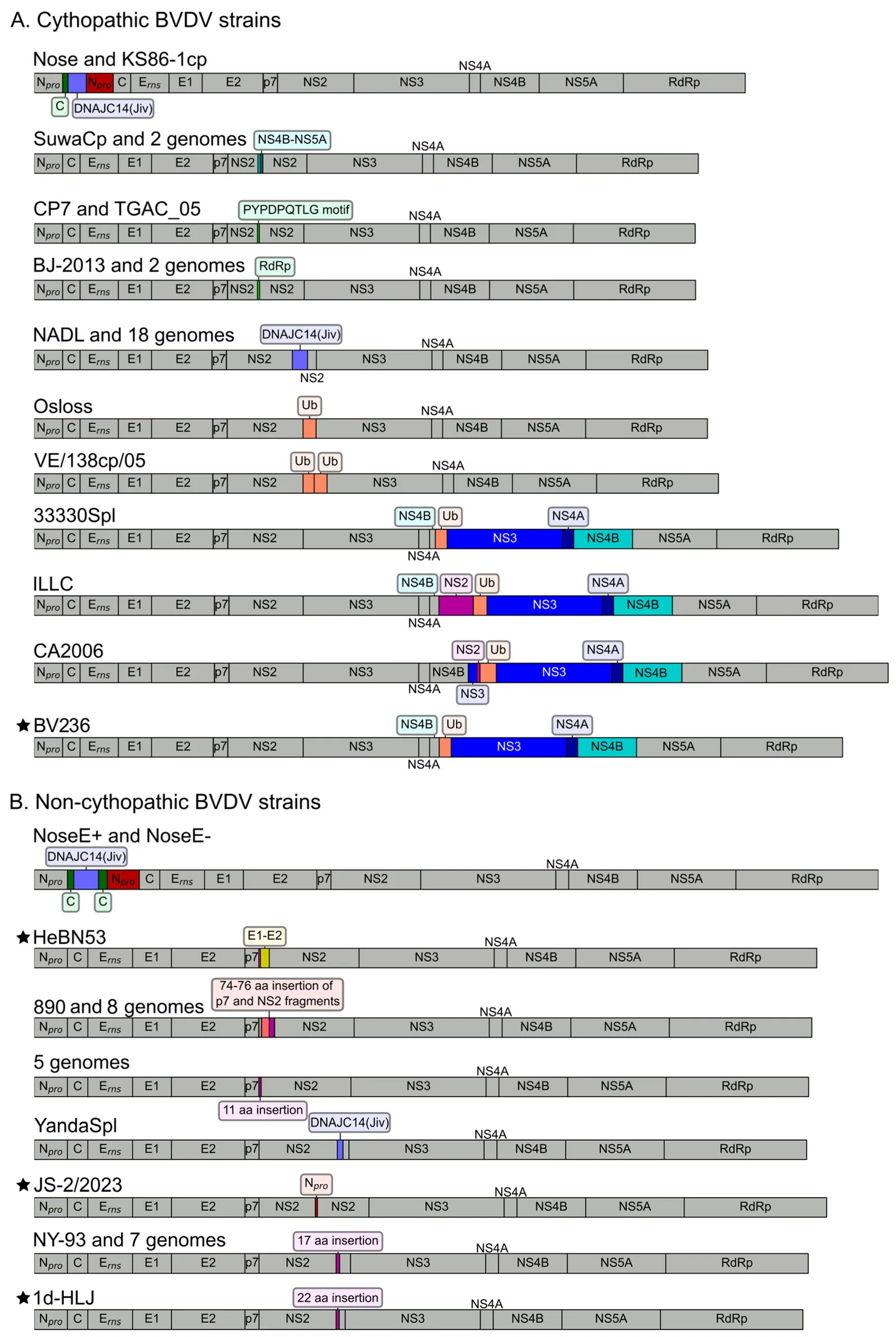
The schematic representation of inserts in cp (**A**) and non-cp (**B**) BVDV isolates with the available complete genome sequence. Viruses isolated after 2015 are indicated by a black star (★). Sequences without a star represent either viruses isolated before 2015 or those for which the isolation year was not explicitly available in the associated GenBank records. The full list of isolates with insertions and their corresponding source references is provided in Table S1.

About a dozen insertions at several loci in the BVDV genome were associated with the cp phenotype [12]. Screening of all available BVDV sequences (N = 566), excluding defective interfering particles (DIPs), such as CP9 [17] and CP13 [18], identified just a few new rearrangements (Figure 1, Table S1). All inserts reportedly associated with the cp biotype occurred at five known hotspots in the genome: the Npro/C junction, the middle of NS2, the 3′-end of NS2, the NS2/NS3 junction, and in the 5′-end of NS4B. Notably, only short inserts (9–28 amino acids) were observed within the middle of the NS2 coding region. Therefore, the number of complete BVDV sequences increased from 59 to 670 over the last 10 years, but the repertoire of known genome rearrangements likely associated with the cp phenotype did not increase accordingly. One novel finding among cp strains was isolate 22NX-9. It was shown in 2021 to produce a cytopathic effect [51], but had no typical insertions associated with the cp phenotype. Following a detailed examination, a non-conservative fragment within the RdRp was detected at polyprotein position 3392–3439, which is unusual, but thus far the only possible explanation for the cp phenotype.

The insertions in the reportedly non-cp BVDV isolates were observed at two of the above hotspots (in the middle of NS2 and 3′ end of NS2), and in an additional hotspot at the p7/NS2 junction or at the 5′-end of NS2, where five distinct insert types ranging from 11 to 76 amino acids were found. The lack of cp effect in genomes with insertions at hotspots correlates with their inability to drive efficient NS2-3 cleavage: the 10-aa Npro fragment lacks a cleavage site, while the 18-aa DNAJC14 protein fragment lacks most of the functional J-domain, probably impairing protease recruitment.

**Figure 2:**
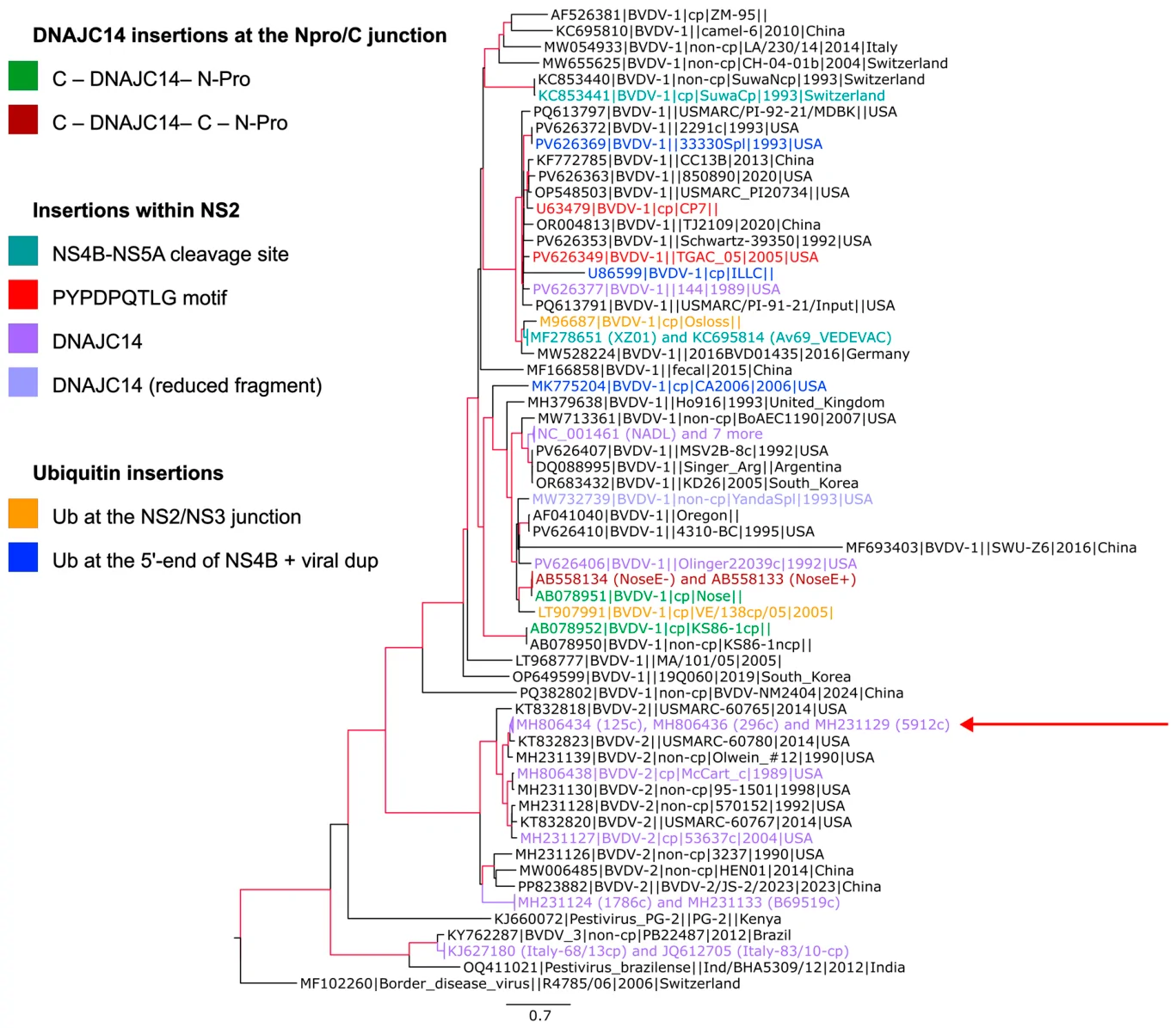
Phylogenetic tree for the reduced dataset of RdRp amino acid sequences of BVDV shows independent emergence of inserts in the cp isolates. Red branches indicate bootstrap support above 0.90 (63% of nodes). The taxon labels are colored according to the type of insertion. The red arrow indicates three near-identical BVDV strains with distinct *DNAJC14* inserts, which are discussed below.

Some insert types were found in a single isolate or in closely related isolates, typically sequenced within the same study (Figures S1–S3). However, several inserts associated with the cp biotype were repeatedly detected in unrelated genomes (Figure 2), with experimental support for cp phenotype effect ranging from direct validation in isolates included in this study to confirmation in strains or constructs carrying similar insertions. These include: the complex *DNAJC14* insert combined with a duplication of C and Npro fragments at the Npro/C junction (two occurrences; supported by experiments with a CP8 isolate [22]); the *DNAJC14* insert at the 3′ end of NS2 (nine independent insertions; validated in [15,52]); the PYPDPQTLG motif within NS2 (two occurrences; confirmed to induce cp in [53]); a ubiquitin-like sequence inserted at the NS2/NS3 junction (two occurrences; validated in [16]); and ubiquitin coupled with a large NS3-NS4A-NS4B duplication in the 5′ part of NS4B (four occurrences; a similar insertion was validated in [54]; its reverse-genetics transfer into CSFV confirmed cp induction [55]). A less well-known insertion—the NS4B-NS5A cleavage site located in the middle of NS2—was observed twice, and its cp effect was validated in a separate isolate carrying similar insertion [56].

Although the ubiquitin-related insertions appear similar, their precise features differ. The ubiquitin-like insertion at the NS2/NS3 junction in strain Osloss is a single copy of ubiquitin, whereas strain VE/138cp/05 contains two copies. The large genome duplication coupled with ubiquitin insertion also has three varieties: strains 33300Spl and BV236 have a duplication of NS3, NS4A, and NS4B; strain ILLC carries an additional NS2 fragment inserted before ubiquitin; and strain CA2006 has an additional short duplication of NS2 and NS3 fragments (Figure 1).

Phylogenetic analysis confirmed that all viruses carrying the insertions described above originated independently (Figure 2). To additionally rule out the possibility that these inserts occurred less frequently and were propagated among BVDV variants by recombination, phylogenies for E1, E2, and RdRp regions (Figures S1–S3) were compared. In all but one case, there was no evidence of recombination that could transfer genome regions bearing insertions and duplications. The only exception to this pattern was observed for the KS86-1cp and Nose strains, both of which carry the *DNAJC14* insert with a duplication of the C and Npro fragments. It was previously shown that the KS86-1cp strain acquired this insert from the Nose strain through homologous recombination, with the crossover occurring precisely at the insertion site [57].

**Figure 3:**
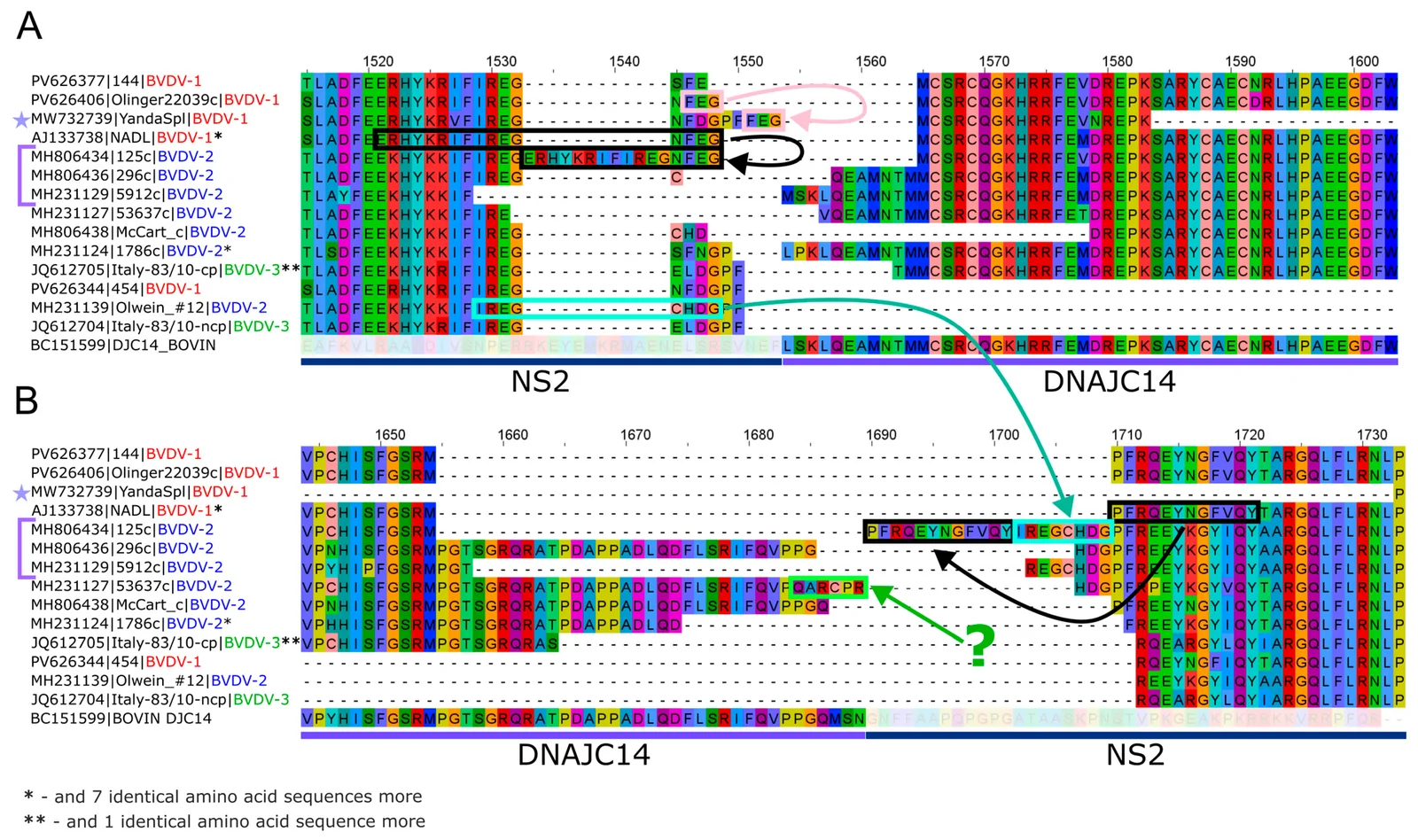
The termini of *DNAJC14* inserts at the 3′ end of NS2 ((**A**)—5′-end of the insertion, (**B**)—3′-end of the insertion). Three genomes of BVDV-1, BVDV-2, and BVDV-3 lacking the *DNAJC14* insertion are shown at the bottom of the alignment as a reference, along with the amino acid sequence of the *Bos taurus* DNAJC14 protein (insertion donor). Within the DNAJC14 sequence shown, the segment that aligns with insertions in viral genomes is colored, whereas the remaining regions are left transparent. Sequences MH806434, MH806436, MH231129 (purple square bracket) have distinct inserts, despite high amino acid sequence similarity (polyprotein identity 97.6%). In BVDV-2 isolate MH806434, regions identical to BVDV-1 reference sequence AJ133738 are marked with black rectangles, and those identical to BVDV-2 isolate MH231139 are marked with a cyan rectangle. Arrows indicate suggestive transfers of amino acid sequences between BVDV-1 and BVDV-2. In isolate MH231127, amino acid positions with no obvious homology to NS2 or DNAJC14 protein are marked with a green rectangle and question mark. The non-cp isolate MW732739 (YandaSpl), which carries an incomplete 19-amino-acid insertion and a partial deletion of NS2, is denoted by a star; a potentially inserted fragment within this isolate is marked with a pink rectangle, with corresponding region in another genome similarly highlighted and connected by an arrow to indicate suggestive similarity. The alignment was visualized in JalView v2.11.5.1 software [37]. The corresponding nucleotide sequence alignment is provided in Figure S4.

Independent emergence of most of the genome rearrangements was further supported by inspection of the nucleotide sequence environment of the insertions. Insertions were considered independent when viruses carrying them (i) grouped separately on phylogenetic trees built using distinct genome regions, or (ii) showed distinct insertion sites and/or junction sequences even when phylogenetically close. For the *DNAJC14* inserts at the 3′-part of NS2, the 5′ and 3′ ends of the inserts differed among the nine independent phylogenetic groups (Figure 3 and Figure S4). When multiple viral sequences were phylogenetically very close and likely represented a single insertion event, they were typically identical across the *DNAJC14* region and its flanking sequences. One exception to this pattern was observed among BVDV-2 isolates MH806434 (USA, 1990), MH806436 (USA, 1995), and MH231129 (USA, 1995) (Figure 2, marked with red arrow; Figure 3, marked with purple square bracket). Although these three isolates grouped together in phylogenetic trees and were highly similar in the viral polyprotein (amino acid identity 97.63%), closer inspection of the alignment revealed that the ends of the *DNAJC14* inserts differed substantially among them, and the precise insertion sites, while close, were not identical (described in detail in Section 3.2 and Figure 3).

**Figure 4:**
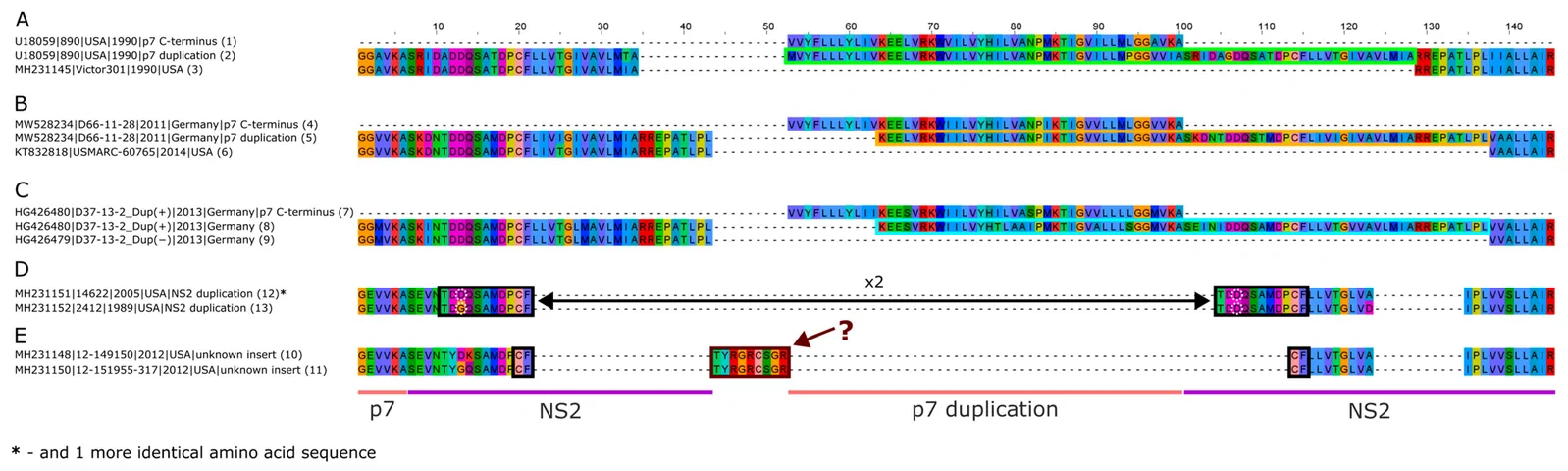
Amino acid sequence alignment of the BVDV polyprotein region flanking the inserts at the 5′-end of NS2 (N-terminal domain of NS2). Panels (**A**–**C**) illustrate three independent duplications of a p7-NS2 fragment. Each event shows, from top to bottom, the p7 C-terminus from the isolate, the NS2 region that contains the duplication (highlighted by a colored rectangle), and the homologous NS2 fragment lacking the insertion from the closest phylogenetic relative. Panels (**D**,**E**) present two additional insertion types at this locus. (**D**) An internal 11-residue tandem duplication within NS2 (black rectangles). (**E**) A 9-aa insertion of unknown origin (brown rectangle) with duplication of two amino acids (CF, black rectangles).

Among the non-cp isolates, only one kind of insertion was repeatedly observed in three phylogenetically unrelated virus groups (Figures S1–S3). These three inserts were 74–76 amino acids long, located at the 5′ end of NS2, and consisted of the 3′-terminus of p7 and the 5′-terminus of NS2. In two of these groups—one consisting of isolate D66-11-28 (GenBank MW528234) and the other of isolate D37-13-2_Dup(+) and related isolates (GenBank HG426480)—the insertion included 37 amino acids from the C-terminus of p7 and 37 amino acids from the N-terminus of NS2, with similar insertion locations (Figure 4A,B). The third type, represented by strain 890, was 76 amino acids long and comprised 48 amino acids from p7 and 28 amino acids from NS2 (Figure 4C). This p7-NS2 duplication appears to be exclusive to BVDV-2. Some of these viruses were shown to be highly pathogenic in naturally and experimentally infected animals but did not demonstrate the cp phenotype [58,59].

Therefore, it is likely that the repertoire of inserts has not been exhaustively explored, but the hotspots for such insertions are certainly limited, because several unrelated insertions were identified at just a few hotspots.

### 3.2. Insertions in the BVDV Genome Are Often Coupled with Duplications of the Viral Genome

Certain cellular gene insertions, including most *DNAJC14* insertions near the 3′ end of NS2 and the insertion of an ubiquitin-like sequence at the NS2/NS3 junction, were consistent with a single recombination event. In such cases, the cellular RNA sequences were exactly flanked by the viral genomic sequences that are normally found at these loci. However, many of the cellular gene acquisitions involved duplications of viral sequences. A typical example is the *DNAJC14* insert between Npro and C that was accompanied by a duplication of Npro and C fragments of 31 and 167 amino acids, respectively (Figure 1).

Independent acquisitions of ubiquitin-like sequences at the 5′-part of NS4B were also coupled with duplications of virus genes, compatible with several recombination events. In strains 33330Spl and BV236, the ubiquitin-like sequence was inserted after truncated NS4B (102 nt and 321 nt, respectively) and was followed by complete duplications of NS3, NS4A, and NS4B (Figure 1). In strain ILLC, the insert was more complex: a 3′-terminal fragment of NS2, followed by the ubiquitin-like sequence and a duplication of NS3, NS4A, and NS4B. The most complex insert was observed in strain CA2006, which retained the longest portion of the original NS4B. This insert comprised a short NS3 fragment (150 nt), followed by a short NS2 fragment (54 nt), the ubiquitin-like sequence, and complete duplications of NS3, NS4A, and NS4B (Figure 1). The precise sequence of events that led to these genomes is unknown, as these rearrangements can be explained by several comparably complex series of insertions, duplications and deletions, but it is clear that they required more than one event.

Among the three phylogenetically related BVDV-2 isolates with a typical *DNAJC14* insertion at the 3′ end of NS2 (Figure 3), one isolate (MH806434, isolated in 1990) had signs of a more complex recombination pattern that involved the acquisition of additional NS2 fragments. This genome contains an additional insertion of the NS2 sequence ERHYKRIFIREG immediately upstream of *DNAJC14* insert (Figure 3A, black rectangle). This sequence is identical at both the amino acid and nucleotide levels to a fragment of NS2 from BVDV-1 strains, whereas in BVDV-2 the sequence E**K**HYK**K**IFIREG is more common at this position. Additionally, a duplication of PFRQEYNGFV is present at the 3′ end of the *DNAJC14* insertion in this genome (MH806434), again identical to BVDV-1 (strain NADL, accession AJ133738), but not to any of the BVDV-2 genomes, in both amino acid and nucleotide sequence (Figure 3B, black rectangles). Notably, this acquisition from BVDV-1 is followed by an additional **IREG** sequence before the remaining NS2 sequence. Together, these features suggest that BVDV-2 isolate MH806434 could have acquired *DNAJC14* together with its flanking NS2 sequences from a BVDV-1 strain in a single recombination event. However, the additional **IREG** sequence found after that insertion points to another non-homologous recombination event. Therefore, “classical” *DNAJC14* inserts in NS2, similarly to larger genome rearrangements coupled with ubiquitin fragment acquisitions, may have evidence of complex recombination patterns.

Even relatively short duplications of the virus genome can be complex. One such example was found within NS2 (Figure 1, strain SuwaCp), where the insertion comprised the NS4B-NS5A protease cleavage site followed by an 11-amino-acid sequence derived from the NS5A downstream from the protease site. In the canonical genomic organization, this 11-aa sequence is located 117 codons downstream of the corresponding protease site. The presence of these two elements, derived from distant genomic positions, suggests that a simple strand-switching mechanism is insufficient to explain this insertion; at least two recombination events would be required.

Another phylogenetic cluster of genomes with variable rearrangements that potentially involved several events included two cp isolates with classical *DNAJC14* inserts (isolates 1786c and B69519c, GenBank accession numbers MH231124 and MH231133, respectively) and five isolates with variable short inserts at the 5′-end of NS2. All these isolates were collected in the USA between 1989 and 2012 [60]. Two isolates, collected in 1989 and 2005, contained a duplicated TD[D/G]QSAMDPC motif from NS2 (Figure 4D). The other three sequences, all collected in the USA in 2012, had the same insertion, TYRGRCSGRCF, in which the terminal CF residues likely represent an NS2 duplication, whereas the origin of the remaining amino acids could not be determined (Figure 4E). This pattern is compatible with a possible loss of a longer insertion over time, leaving behind these shorter NS2-derived sequences.

Collectively, these examples illustrate that variable insertions at different locations in the BVDV genome involve duplications of viral sequence and thus are indicative of multiple recombination events.

### 3.3. Insertions in BVDV Genomes Can Be Long or Short, but Rarely Intermediate

**Figure 5:**
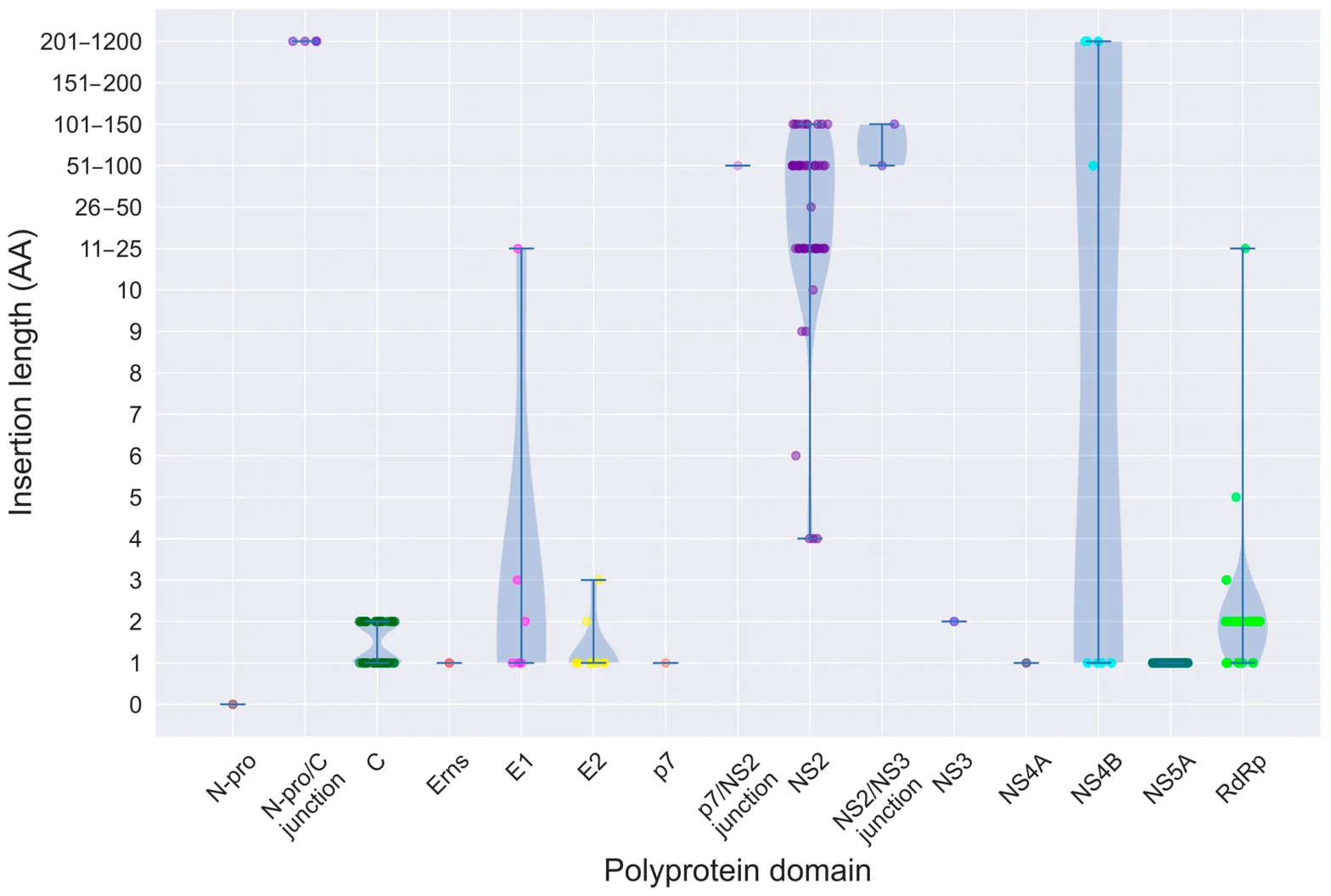
Insertion length distribution across the BVDV polyprotein. Each dot represents an insertion in a single polyprotein and is colored according to insertion location (mature protein). The width of each violin (shaded area) represents the insertion length density at different values.

In addition to larger insertions (more than 11 amino acids), there were multiple short insertions in BVDV genomes. The number of such insertions varied among the BVDV proteins, but their length was almost always 1–2 amino acids (Figure 5). This contrasted with long inserts that are usually linked to the cp biotype, which were at least 9 amino acids long (e.g., a 27 nt insertion in cp CP7 strain [61]), but often over 50 amino acids. Notably, there were generally no short 1–2 aa inserts at the sites of long inserts, and short insertions (<9 aa) were generally uncommon in NS2 (four inserts per 452 amino acids in NS2 vs. 307 inserts per 3444 aa in the rest of the genome, one-sided Fisher’s exact test, odds ratio = 0.091, *p*-value = 7.05 × 10^−13^), although NS2 is a hotspot for long insertions (Figure 5). Therefore, NS2 is prone only to long, but not short insertions. The pattern is compatible with different mechanisms of short and long insertions.

### 3.4. Insertions Can Be Reverted by Distinct Mechanisms

The cp biotype is usually considered an ecological dead-end, because these viruses replicate to high levels and cause severe disease in a persistently infected host, usually resulting in a rapid death, but are not capable of sustained circulation among animals that are not persistently infected with a non-cp variant. Prolonged circulation of the cp biotype has been described among animals that are persistently infected by the non-cp biotype [62]. However, it was not clear whether the cp viruses can revert to the non-cp phenotype and return to a classical circulation mode. We screened the full-genome phylogenetic tree of all publicly available BVDV sequences (Figure S1) for evidence of phenotype reversion and found two potential instances.

**Figure 6:**
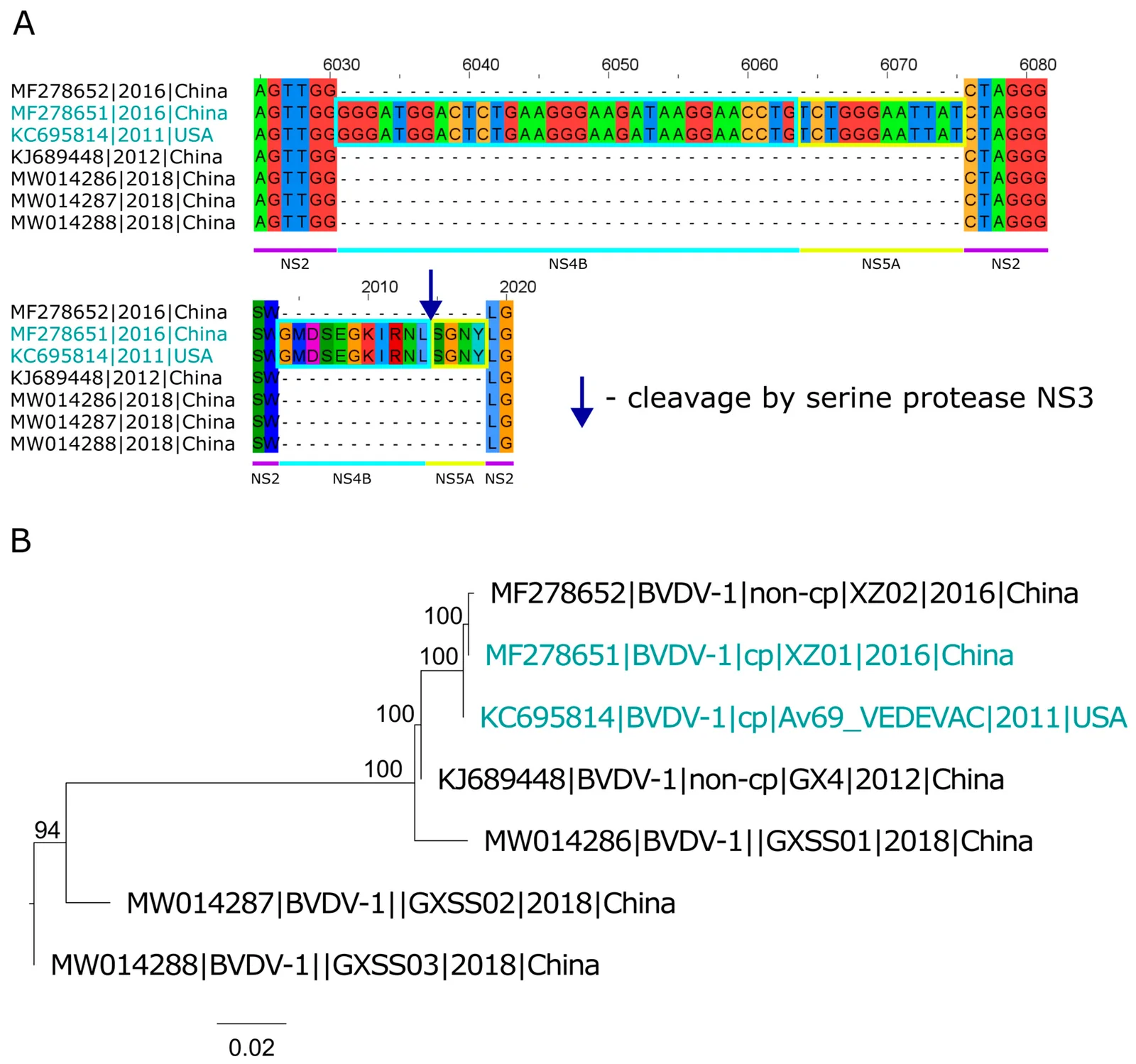
Phylogenetic analysis of isolates harboring the NS4B-NS5A protease cleavage site insert in NS2 suggests a descendant with insertion loss. (**A**) Nucleotide (**upper panel**) and amino acid (**lower panel**) sequence alignments of the insertion and the flanking NS2 regions. The inserted sequence is marked with cyan and yellow boxes (NS4B and NS5A, respectively); the protease cleavage site is indicated by a blue arrow on the amino acid alignment. (**B**) ML phylogenetic tree inferred from the RdRp nucleotide sequences using IQ-TREE v3.0.1. Isolates with the insertion are denoted by cyan labels.

A possible loss of the cp insert was detected in a Chinese non-cp isolate MF278652 collected in 2016 [63]. This virus has a cp peer MF278651 (China, 2016) that has a unique insert of the NS4B-NS5A cleavage site within NS2 [64] and otherwise nearly identical genomic sequence. These two viruses could present a typical non-cp/cp virus pair. However, GenBank genome KC695814 (collection location USA, 2011, sequenced in China, according to the GenBank record) is an outgroup of these two viruses on a phylogenetic tree and has the same insertion in NS2 (Figure 6). It is unlikely that the same rare insert was acquired independently in two non-cp viruses, and it is thus more likely that a descendant of KC695814 was circulating as a cp strain and lost its insert subsequently, yielding MF278652.

A likely sign of incomplete *DNAJC14* loss is seen in strain YandaSpl (GenBank accession MW732739) (Figure 3, denoted with a star). This non-cp isolate (according to the Genbank record) retained 19 amino acids of DNAJC14 protein but lacked 21 amino acids of NS2 at the corresponding genome positions. Therefore, it was likely recovered from a typical cp genome by losing the *DNAJC14* insert while maintaining almost precise length of the original NS2, but not the sequence. Alternatively, this could be a failed insertion of a short *DNAJC14* fragment. However, such a scenario would not explain the survival of this virus and would be less compatible with the known patterns of BVDV evolution.

There were also two examples of insertion loss that have been suggested previously. An insertion loss in a highly pathogenic (but non-cp) BVDV was observed among isolates from an outbreak in Germany in 2013 [65]. This set of genomes included multiple variants with and without a 222 nt duplication of the p7-NS2 fragment (Figure 4C, isolate D37-13-2_Dup(+)). Using a reverse genetic system with a strain 890 (Figure 1) harboring a similar insert with p7 and NS2 fragments, it has been experimentally demonstrated that during replication, the genome without duplication is generated de novo from the genome with a duplication through a deletion event [65].

**Figure 7:**
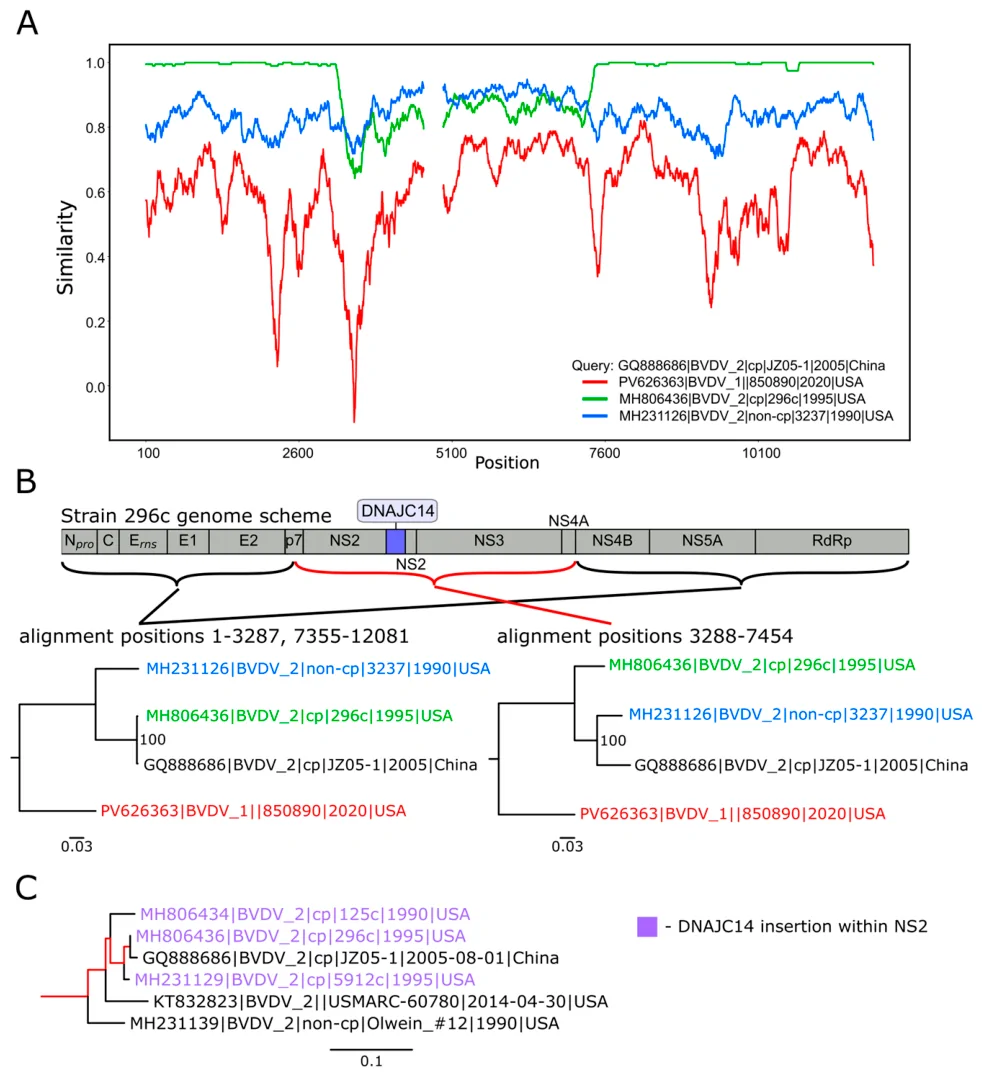
Loss of *DNAJC14* insert in the NS2 region via homologous recombination. (**A**) Simplot analysis of the recombinant isolate JZ05-1 (GQ888686). (**B**) Schematic of the strain 296c genome and ML phylogenetic trees for genome regions indicated by braces. Bootstrap support values are shown at the tree nodes. Taxa labels are color-coded as in panel A. (**C**) A fragment of the ML tree for RdRp nucleotide sequence shows the position of JZ05-1 among its closest genetic relatives. Isolates that retain the *DNAJC14* insert are denoted by purple labels. Red tree branches are supported by ultrafast bootstrap values > 90%.

There was also evidence that the non-cp genome can be recovered by homologous recombination with an unrelated BVDV genome. Isolate JZ05-1 (GQ888686), isolated from cattle in China in 2005, was reportedly cp [66], but its genome does not contain any known modifications compatible with the cp biotype. Previously, it was identified as a putative recombinant [67,68]. Our analysis of recombination on the updated dataset of BVDV complete genomes showed that the majority of the JZ05-1 genome indeed has >99% nucleotide identity with a clade of cp isolates represented by USA strains, while p7, NS2, NS3, and NS4A come from an unknown minor parent that does not contain *DNAJC14* insert (Figure 7A). Unlike the resolution of NRR-generated duplications, which involves recombination between closely related genomes, this recombination event occurred with an unrelated genome, and the cross-over points are located distant from the insertion site. This observation provides the first evidence that homologous recombination can mediate the deletion of the *DNAJC14* insertion. This was not apparent in an earlier study, which was conducted on a smaller subset of complete BVDV genomes [67].

## 4. Discussion

The phenomenon of large inserts in the BVDV genome is a key factor in the pathogenesis of lethal mucosal disease [16]. The repertoire of inserts is relatively large, and some genome rearrangements have repeatedly emerged in independent virus lineages (Figure 2). The molecular mechanism underlying these acquisitions remains unknown, although it has been hypothesized that this is driven by non-replicative recombination that promiscuously joins viral and cellular RNA fragments [12]. However, the option of non-homologous replicative recombination (polymerase switching between template RNA strands) could also be considered.

Although replicative recombination is usually homologous, it can also be illegitimate, i.e., the polymerase can switch between non-homologous templates and yield chimeric genomes. This was demonstrated by experiments on poliovirus polymerase that resolved genome duplications by illegitimate recombination more often than by legitimate [30]. However, replicative recombination does not explain why many cellular RNA acquisitions in BVDV were coupled with the duplication of viral genes. Hypothetically, viral polymerase can become strand switching-prone and more capable of multiple illegitimate recombination events under certain conditions, as was demonstrated experimentally in the presence of antiviral drug ribavirin [30]. Therefore, it is conceivable that this occurs in some cells and leads to production of multi-chimeric genomes. However, replicative recombination does not readily explain why duplications and cellular RNA acquisitions in BVDV are at least 27 nt long. The distribution of indels in BVDV genomes is strikingly binomial, with many inserts of 3–6 nucleotides (1–2 amino acids), many duplications and insertions above 30 nt (10 aa), but few events of intermediate length (Figure 5). RdRp of orthoflaviviruses (a genus of the same family *Flaviviridae* as pestiviruses) shifts between priming and elongation conformations [69]. Suggesting that there are also minimum elongation length requirements on a new template, this could explain minimum insert length, but there is no experimental evidence for this hypothesis. Therefore, replicative recombination might explain the external gene acquisitions in BVDV genomes, but this would require several speculative assumptions.

Non-replicative recombination (NRR) appears to be a more likely mechanism of BVDV genome rearrangements, as has been suggested previously [12]. NRR is a well-established phenomenon in several viruses, but its mechanisms are not clear. The best-known RNA cutting and ligation system in a cell is involved in the tRNA metabolism. However, tRNA cutters (both ribozymes and endonucleases) have a high specificity to target sequences, while NRR was experimentally verified in several viruses and at many locations within viral genomes [27,28,29,30,31]. Also, non-replicative recombination has been shown to have RNA end phosphorylation requirements (discussed below) that are not compatible with tRNA ligases [31].

A mechanism that could explain the observed pattern of large genomic rearrangements in BVDV is non-replicative recombination by endonuclease IRE1 and RtcB ligase. These enzymes were originally discovered as a part of unfolded protein response (UPR) and act as the core of the machinery that mediates unconventional splicing of XBP1 mRNA, which then upregulates chaperone genes, enhancing ER folding capacity. Several points make this explanation possible.

1. IRE1 [70] and human RtcB [71] are associated with the ER, which is also the BVDV replication site [72]. Thus, two enzymes and viral RNA are sterically close to each other. Moreover, there is direct evidence that IRE1 is recruited to the replication site of Zika virus, another member of the family *Flaviviridae* [73].

2. IRE1 was originally believed to be highly specific for its role in XBP1 splicing. However, it was recently shown to degrade RNA non-specifically as a part of a non-canonical mRNA decay pathway termed RIDDLE [74]. Although there is no direct evidence that RIDDLE acts in bovine cells in the same way, it appears possible, because ER and RNA metabolism are among the basic cellular functions. Therefore, it is conceivable that RIDDLE produces a mixture of cellular and viral RNA fragments that are subsequently ligated by RtcB. This explains common coupling of genome duplications with cellular gene acquisitions in BVDV.

3. RtcB ligase joins RNA with a 2′-3′ cyclic phosphate or 3′-phosphate to a 5′OH acceptor [75], in contrast to canonical RNA ligases requiring 3′OH and 5′ phosphate ends. To date, this is the only known ligase that can process such RNA ends [76]. This RNA end phosphorylation pattern was necessary for experimental non-replicative recombination in poliovirus to produce end-to-end ligation of RNA fragments [31]. Notably, another study on non-replicative RNA recombination used RNA transcripts produced by T7 or SP6 polymerases, which yield 3′OH ends that are not suitable for RtcB [77]. In that study, no precise end-to-end fragment joining was observed, suggesting that the transfected RNAs had to be first processed by a compatible endonuclease, such as IRE1.

**Figure 8:**
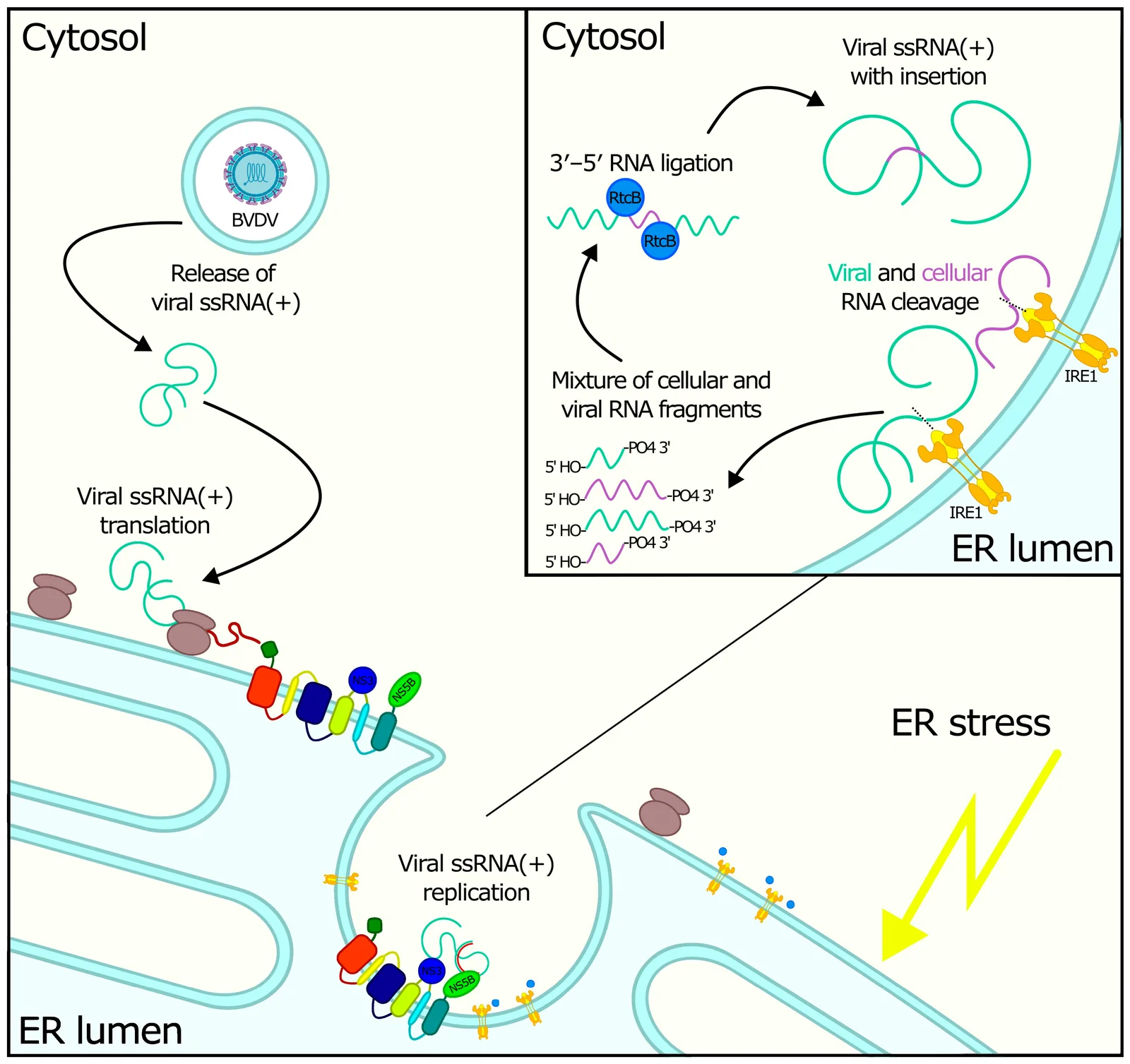
The proposed role of cellular endonuclease IRE1 and 3′-5′ ligase RtcB in BVDV genome rearrangements. BVDV forms a virus replication compartment on the ER membrane. ER stress induces IRE1 and RtcB, both of which interact with viral and host RNAs. IRE1 cleaves RNA while RtcB mediates 3′-5′ ligation.

Therefore, cellular function, steric location, insert length and end phosphorylation requirements are all consistent with potential involvement of endonuclease IRE1 and RtcB ligase (or their close homologs/paralogs) in generation of cellular RNA acquisitions in the BVDV genome and a non-replicative recombination mechanism in general. The hypothetical mechanism would thus be the following (Figure 8). Unfolded protein stress occurs in certain cells with a replicating BVDV (not necessarily as a consequence of the virus replication). IRE1 processes cellular mRNA and viral RNA to produce a mix of fragments. It is likely that the repertoire of fragments is limited by both abundance of particular RNAs in proximity to the ER and the preferred IRE1 digestion patterns, because even non-specific RNA processing by IRE1 (RIDDLE) produced a ladder of fragments rather than a smear [74]. This could help explain the finite number of genome rearrangements observed in BVDV, although the fitness gain from inserts (only replication-competent cp phenotype can outcompete the original non-cp genome) and the limitations from virus protein integrity (inserts are both tolerable and beneficial only at a few loci) might be more important factors here. The RNA fragments produced by IRE1 from viral and cellular RNA have identical ends, 2′-3′cyclic phosphate at the 3′ end and 5′-OH. They are subsequently ligated in a random manner by RtcB, which is present at the same location. This explains why inserts in BVDV are often coupled with genome duplications. The concept of such a pool of processed RNA pieces as a source of fragments for non-replicative recombination is further supported by a report that XRN1 exoribonuclease can suppress recombination in Tomato bushy stunt virus in yeast by depleting a pool of uncapped RNA fragments (i.e., with a free 5′-OH) in a cell [78].

At this stage, this hypothesis has only fragmentary support from experimental data. Further studies might include generation of knockout or overexpressing cell lines (e.g., by CRISPR-Cas) and testing NRR efficiency depending on the availability of hypothetical actors.

Regardless of the illegitimate recombination mechanism, it is conceivable that the chimeric genomes are subsequently processed by replicative recombination, which had been experimentally shown to resolve viral RNA (delete excess inserts) after non-replicative recombination [30]. Only viable genomes could be selected and sequenced as natural isolates. This sequence of events (NRR, resolution by replicative recombination and purification by selection) makes it almost impossible to infer actual sites and patterns of the original NRR. We tried to analyze genome regions that were adjacent to the inserts but did not observe any consistent pattern. Instead, there was a strict requirement for insertions to be at a few permissive sites in the genome. It is conceivable that subsequent selection for functional viral proteins had a greater effect on the observed recombination pattern than the mechanism of the initial recombination.

NRR was experimentally confirmed in poliovirus and hepatitis C virus. Although these viruses recruit the ER membrane (and thus, hypothetically, IRE1 and RtcB) to their replication complexes [79,80], in the NRR experiments viral RNA had not been translated yet [27,77]. Thus, some level of NRR might occur in any virus. However, experimental NRR was a rare event. In a typical experiment, 10^7^ cells are transfected (thus severely stressing the cells and likely inducing UPR) with 10^11^–10^12^ RNA molecules to yield just tens of viable virus plaques. It is likely that long-term persistence of BVDV increases chances of NRR, but overall, there is no evidence that NRR is a very common event, in contrast to the replicative homologous recombination [81]. This observation is relevant for predicting evolution of RNA viruses and emergence of novel biological threats. On the one hand, any combination of cellular and viral RNA can theoretically emerge at any moment by NRR. On the other hand, such successful acquisitions in RNA virus genomes other than BVDV are scarce and are usually associated with the emergence of novel virus genera. In this respect, BVDV is a convenient model that reproduces exceptionally rare evolutionary events on a measurable scale.

A major limitation of this study stems from the use of isolates with complete coding sequences available, thereby excluding a substantial number of sequences for which only partial genomic information exists. Notably, several cp pestivirus sequences harboring cellular insertions described in the literature—including those derived from GATE-16, LC3, NEDD8, GABA(A)-RAP, and SMT3B mRNA—are represented in GenBank exclusively by PCR products and were therefore not amenable to detailed analysis. The defective interfering particles (DIPs) were also omitted from the dataset. Despite these constraints, the screening of available full genomes provided a suitable framework for investigating the evolutionary patterns of insertions in BVDV.

Another limitation of the study is reliance on publicly available genomes that may vary in sequencing and annotation quality. While the sum of evidence supports the claims, some of the individual examples may be questioned for reasons of raw data integrity.

## Data Availability

The data presented in this study (sequence names and metadata, alignments, phylogenetic trees) are openly available at https://github.com/WizardFPS/Systematic-analysis-of-insertions-across-pestivirus-genomes (accessed on 6 July 2026).

## Supplementary Materials

The following supporting information can be downloaded at https://www.mdpi.com/article/10.3390/v18080886/s1, Table S1: Location of insertions in the BVDV genome; Figure S1: Maximum likelihood phylogenetic tree for RdRp nucleotide sequence built using FastTree for all available complete genome sequences of BVDV. The tree was rooted by the outgroup (Border disease virus; GenBank accession numbers: MF102260, MF102261); Figure S2: Maximum likelihood phylogenetic tree for E1 nucleotide sequence built using FastTree for all available complete genome sequences of BVDV. The tree was rooted by the outgroup (Border disease virus; GenBank accession numbers: MF102260, MF102261); Figure S3: Maximum likelihood phylogenetic tree for E2 nucleotide sequence built using FastTree for all available complete genome sequences of BVDV. The tree was rooted by the outgroup (Border disease virus; GenBank accession numbers: MF102260, MF102261); Figure S4: Variation of the termini of *DNAJC14* insert at the 3′ end of NS2 ((A)—5′-end of the insertion, (B)—3′-end of the insertion). Three genomes of BVDV-1, BVDV-2, and BVDV-3 lacking the *DNAJC14* insertion are shown at the bottom of the alignment, along with the nucleotide sequence of mRNA encoding the *Bos taurus* DNAJC14 protein (*DNAJC14* gene fragment donor). Sequences MH806434, MH806436, MH231129 (purple square bracket) have distinct inserts, despite high amino acid sequence similarity (polyprotein identity 97.63%). In isolate MH806434, regions identical to BVDV-1 reference AJ133738 are marked with black rectangles, and those identical to BVDV-2 isolate MH231139 are marked with a cyan rectangle. Arrows indicate suggestive transfers of amino acid sequences between BVDV-1 and BVDV-2. In isolate MH231127, nucleotides with no obvious homology to NS2 or *DNAJC14* are marked with a green rectangle. The non-cytopathogenic isolate MW732739 (YandaSpl), which carries an incomplete 19-amino-acid insertion of *DNAJC14* and a partial deletion of NS2, is denoted by a star. The alignment was visualized in JalView v2.11.5.1 software [46]. References [82,83,84,85,86,87,88,89,90,91,92] are cited in Table S1.

## Abbreviations

The following abbreviations are used in this manuscript:

BVDV	bovine viral diarrhea virus
cp	cytopathogenic
DIP	defective interfering particle
DNAJC14	DnaJ homolog subfamily C member 14
IRE1	inositol requiring enzyme 1
ML	maximum likelihood
mPTP	mitochondrial permeability transition pore
non-cp	non-cytopathogenic
NRR	non-replicative recombination
RDP4	Recombination Detection Program version 4
RIDDLE	regulated IRE1-dependent decay lacking endomotif
RtcB	RNA 2′,3′-cyclic phosphate and 5′-OH ligase
UPR	unfolded protein response
VDAC-1	voltage-dependent anion channel 1
