# Supplementary material for: Comprehensive Analysis of Insertions in BVDV and Implications for Non-Homologous Recombination Mechanisms
Source: Viruses. 2026 Aug 12;18(8):886. doi: 10.3390/v18080886 (PMC13517609; doi:10.3390/v18080886)
Supplement: Supplementary file 1 [file viruses-18-00886-s001.zip › FigureS2_BVDV_E1_fasttree.pdf]

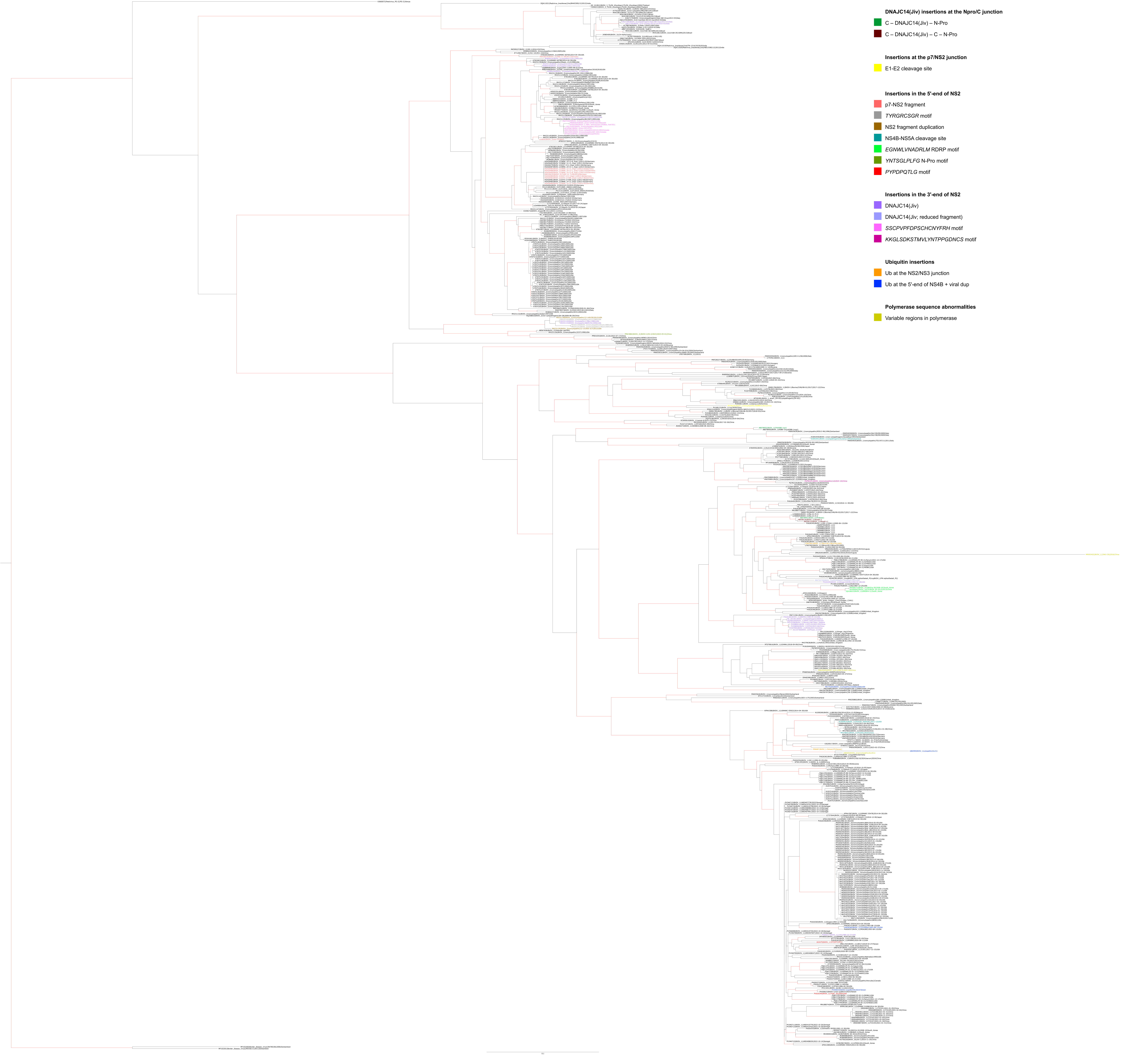
DNAJC14(Jiv) insertions at the Npro/C junction
C – DNAJC14(Jiv) – N-Pro
C – DNAJC14(Jiv) – C – N-Pro
Insertions at the p7/NS2 junction
E1-E2 cleavage site
Insertions in the 5'-end of NS2
p7-NS2 fragment
TYRGRCSGR motif
NS2 fragment duplication
NS4B-NS5A cleavage site
EGNWLVNADRLM RDRP motif
YNTSGLPLFG N-Pro motif
PYPDPQTLG motif
Insertions in the 3'-end of NS2
DNAJC14(Jiv)
DNAJC14(Jiv; reduced fragment)
SSCPVPFDPSCHCNYFRH motif
KKGLSDKSTMVLYNTPPGDNCS motif
Ubiquitin insertions
Ub at the NS2/NS3 junction
Ub at the 5'-end of NS4B + viral dup
Polymerase sequence abnormalities
Variable regions in polymerase
