# Supplementary material for: Comprehensive Analysis of Insertions in BVDV and Implications for Non-Homologous Recombination Mechanisms
Source: Viruses. 2026 Aug 12;18(8):886. doi: 10.3390/v18080886 (PMC13517609; doi:10.3390/v18080886)
Supplement: Supplementary file 1 [file viruses-18-00886-s001.zip › FigureS4_jiv90_nt_alignment.pdf]

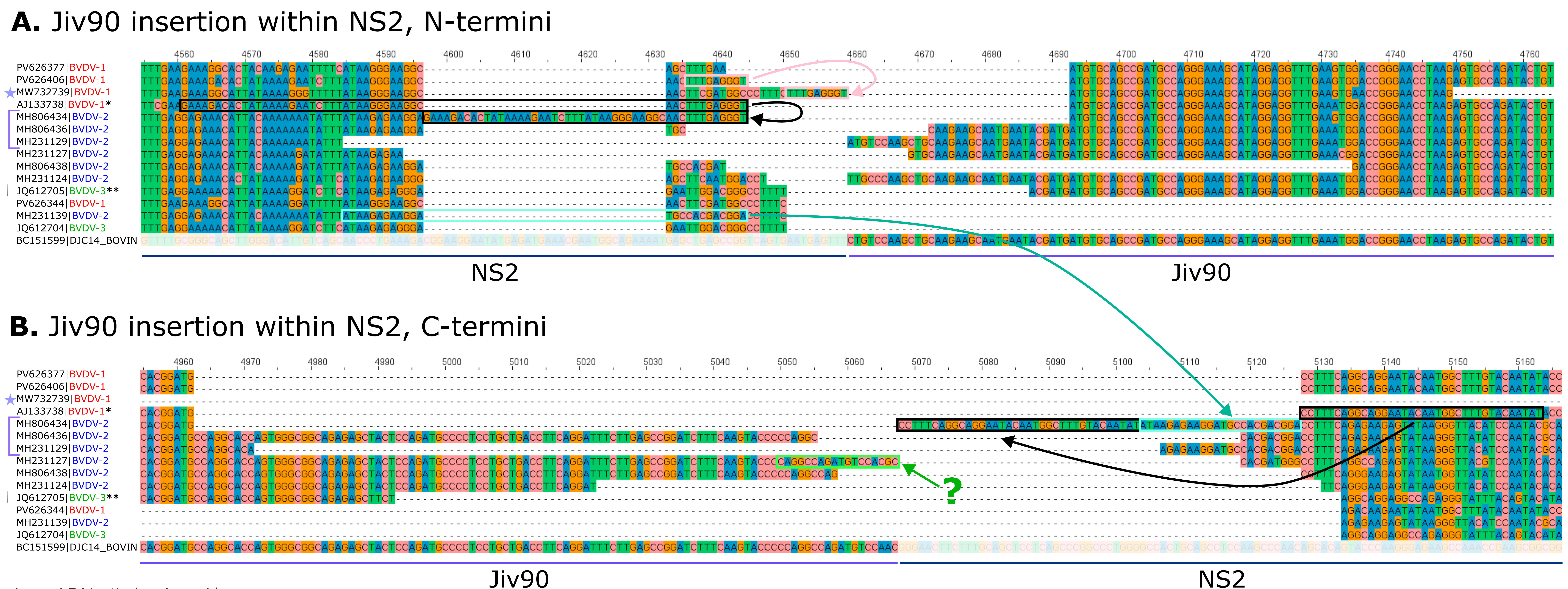

* - and 7 identical amino acid sequences more

** - and 1 identical amino acid sequence more
