# Supplementary material for: Comprehensive Analysis of Insertions in BVDV and Implications for Non-Homologous Recombination Mechanisms
Source: Viruses. 2026 Aug 12;18(8):886. doi: 10.3390/v18080886 (PMC13517609; doi:10.3390/v18080886)
Supplement: Supplementary file 1 [file viruses-18-00886-s001.zip › TableS1.pdf]

Table S1. Genomic locations of insertions in BVDV strains

Location of insertion	Inserted sequence	Genbank accession (strain)	Biotype	Isolation year	Reference
Npro/C	C, Jiv90, Npro	1 AB078951 (**Nose**)	cp	ND	[1]
		AB078952 (KS86-1cp)	cp	ND	
	C, Jiv90, C Npro	AB558133 (NoseE+)	non-cp	ND	Direct submission
		AB558134 (NoseE-)	non-cp	ND	Direct submission
p7/NS2	E1-E2 protease cleavage site	PP706246 (HeBN53)	non-cp	2023	Direct submission
Beginning of NS2	76 aa insertion with p7-NS2 fragment	U18059 (890)	non-cp	ND	[2]
	11 insertion with AMDPCF motif	MH231148 (12-149150)	non-cp	2012	Direct submission
		MH231149 (Short)	non-cp	1989	Direct submission
		MH231150 (12-151955-317)	non-cp	2012	Direct submission
		MH231151 (14622)	non-cp	2005	Direct submission
		MH231152 (2412)	non-cp	1989	Direct submission
	74 aa insertion with p7-NS2 fragment	MW528235 (TV02-13_T136)	ND	2013	[3]

		HG426480 (D37-13-2_Dup(+))	non-cp	2013	[4]
		HG426482 (D75-13-609_Dup(+))	ND	2013	
		HG426484 (NRW_12-13_Dup(+))	ND	2013	
		HG426486 (NRW_14-13_Dup(+))	ND	2013	
		HG426488 (NRW_19-13-1_Dup(+))	ND	2013	
		HG426490 (NRW_19-13-8_Dup(+))	ND	2013	
	74 aa insertion with p7-NS2 fragment	MW528234 (D66-11-28)	ND	2011	[3]
Within NS2	*protease cleavage site of NS4B-NS5A	KC853441 (SuwaCp)	cp	1993	[5]
		MF278651 (XZ01)	cp	2016	[6]
		KC695814 (Av69_VEDEVAC)	cp	2011	Direct submission
	12 aa RDRP fragment	MH490942 (BJ-2013)	cp	2013	[7]

	MK509773 (BVD1a-JB)	ND	2008	Direct submission
	JQ418633 (08GB44-1)	ND	ND	Direct submission
*9-11 aa insertion with PYPDPQTLG motif	PV626349 (TGAC_05)	ND	2005	[8]
	U63479 (CP7)	cp	ND	[9,10]
10 aa Npro fragment	PP823882 (JS-2/2023)	ND	2023	Direct submission
*Jiv90	PV626384 (88055c)	ND	1988	[8]
	PX275358 (Bovax)	ND	1967	[11]
	PV207994 (PZ20)	ND	2023	Direct submission
	PV208006 (PZ54)	ND	2023	Direct submission
	PV208001 (PZ132)	ND	2023	Direct submission
	AJ133738 (**NADL**)	cp	1988	[12]
	NC_001461 (NADL)	cp	1988	[13]
	OQ784258 (NADL-VIEV)	ND	2023	Direct submission
	PV626406 (Olinger22039c)	ND	1992	[8]
	PV626377 (144)	ND	1989	[8]
	MH806438 (McCart_c)	cp	1989	[14]

	MH231127 (53637c)	cp	2004	
	MH806434 (125c)	cp	1990	
	MH231129 (5912c)	cp	1995	
	MH806436 (**296c**)	cp	1995	
	MH231124 (1786c)	cp	1989	
	MH231133 (B69519c)	cp	2006	
	KJ627180 (Italy-68/13cp)	cp	2013	[15]
	JQ612705 (Italy-83/10-cp)	cp	2010	[16]
22 aa insertion with Jiv90 and deletion in NS2	MW732739 (YandaSpl)	non-cp	1993	[17]
17 aa insertion (SSCPMPFDPS CHCNYFR)	HQ174303 (37621)	non-cp	ND	[18]
	HQ174302 (793)	non-cp	ND	
	HQ174301 (IAF-103)	non-cp	ND	
	KR093034 (NY-93)	non-cp	1993	[18,19]
	KP057803 (24515)	non-cp	1993	[18,19]
	AY149216 (p24515)	non-cp	ND	Direct submission

		AF145967 (1373)	non-cp	ND	[18,19]
		AF502399 (NY'93/C)	non-cp	1993	[20]
	22 aa insertion KKGLSDKST MVLYNT	PQ782221 (1d-HLJ)	non-cp	2024	Direct submission
NS2/NS3	*ubiquitin domain	LT907991 (VE/138cp/05)	cp	2005	Direct submission
		M96687 (Osloss)	cp	1965	[21]
NS4A/NS4B	ubiquitin, NS3, NS4A, NS4B	PV626369 (33330Spl)	ND	1993	[8]
	ubiquitin, NS3, NS4A, NS4B	PV426616 (BV236)	cp	2023	[22]
	NS2, ubiquitin, NS3, NS4A, NS4B	U86599 (ILLC)	cp	ND	Direct submission
	NS3 (fragment), NS2 (short fragment), ubiquitin, NS3, NS4A, NS4B	MK775204 (CA2006)	cp	2006	Direct submission

* Insertions that occurred several times independently are marked with asterisks
ND, no data

References

1. Nagai, M.; Sakoda, Y.; Mori, M.; Hayashi, M.; Kida, H.; Akashi, H. Insertion of Cellular Sequence and RNA Recombination in the Structural Protein Coding Region of Cytopathogenic Bovine Viral Diarrhoea Virus. *J. Gen. Virol.* **2003**, *84*, 447–452, doi:10.1099/vir.0.18773-0.

2. Ridpath, J.F.; Bolin, S.R. The Genomic Sequence of a Virulent Bovine Viral Diarrhea Virus (BVDV) from the Type 2 Genotype: Detection of a Large Genomic Insertion in a Noncytopathic BVDV. *Virology* **1995**, *212*, 39–46, doi:10.1006/viro.1995.1451.

3. King, J.; Pohlmann, A.; Dziadek, K.; Beer, M.; Wernike, K. Cattle Connection: Molecular Epidemiology of BVDV Outbreaks via Rapid Nanopore Whole-Genome Sequencing of

Clinical Samples. *BMC Vet. Res.* **2021**, *17*, 242, doi:10.1186/s12917-021-02945-3.

4. Jenckel, M.; Höper, D.; Schirrmeier, H.; Reimann, I.; Goller, K. V.; Hoffmann, B.; Beer, M. Mixed Triple: Allied Viruses in Unique Recent Isolates of Highly Virulent Type 2 Bovine Viral Diarrhea Virus Detected by Deep Sequencing. *J. Virol.* **2014**, *88*, 6983–6992, doi:10.1128/JVI.00620-14.

5. Marques Antunes de Oliveira, A.; Stalder, H.; Peterhans, E.; Sauter, K.-S.; Schweizer, M. Complete Genome Sequences of Both Biotypes of a Virus Pair of Bovine Viral Diarrhea Virus Subgenotype 1k. *Genome Announc.* **2013**, *1*, doi:10.1128/genomeA.00287-13.

6. Luo, R.; Wei, X.; Zhou, H.; Sizhu, S. Characterization of Two Bovine Viral Diarrhea Virus Strains Originating from Cattle in Tibet, China. *Genome Announc.* **2017**, *5*, doi:10.1128/genomeA.01087-17.

7. Liu, C.; Villanova, F.; Leal, É.; Liang, L.; Zhang, Y.; Cui, S. Characterization and Evolutionary Analyses of Pestivirus A Isolated from Cattle in China 2019.

8. Holthausen, D.J.; Bayles, D.O.; Neill, J.D.; Dassanayake, R.P.; Falkenberg, S.M.; Menghwar, H.; Casas, E. Deletion Viral Genome Diversity among Bovine Viral Diarrhea Virus (BVDV) 1a and 1b Strains. *Virol. J.* **2025**, *22*, 237, doi:10.1186/s12985-025-02773-z.

9. Meyers, G.; Tautz, N.; Becher, P.; Thiel, H.J.; Kümmerer, B.M. Recovery of Cytopathogenic and Noncytopathogenic Bovine Viral Diarrhea Viruses from CDNA Constructs. *J. Virol.* **1996**, *70*, 8606–8613, doi:10.1128/jvi.70.12.8606-8613.1996.

10. Tautz, N.; Meyers, G.; Stark, R.; Dubovi, E.J.; Thiel, H.J. Cytopathogenicity of a Pestivirus Correlates with a 27-Nucleotide Insertion. *J. Virol.* **1996**, *70*, 7851–7858, doi:10.1128/jvi.70.11.7851-7858.1996.

11. Dunowska, M.; Lal, R.; Dissanayake, S.; Bond, S.; Burrows, E.; Moffat, J.; Howe, L. Bovine Viral Diarrhoea Viruses from New Zealand Belong Predominantly to the BVDV-1a Genotype. *N. Z. Vet. J.* **2024**, *72*, 66–78, doi:10.1080/00480169.2023.2291039.

12. Vassilev, V.B.; Donis, R.O. Bovine Viral Diarrhea Virus Induced Apoptosis Correlates with Increased Intracellular Viral RNA Accumulation. *Virus Res.* **2000**, *69*, 95–107, doi:10.1016/S0168-1702(00)00176-3.

13. Collett, M.S.; Larson, R.; Gold, C.; Strick, D.; Anderson, D.K.; Purchio, A.F. Molecular Cloning and Nucleotide Sequence of the Pestivirus Bovine Viral Diarrhea Virus. *Virology* **1988**, *165*, 191–199, doi:10.1016/0042-6822(88)90672-1.

14. Neill, J.D.; Workman, A.M.; Hesse, R.; Bai, J.; Porter, E.P.; Meadors, B.; Anderson, J.; Bayles, D.O.; Falkenberg, S.M. Identification of BVDV2b and 2c Subgenotypes in the United States: Genetic and Antigenic Characterization. *Virology* **2019**, *528*, 19–29, doi:10.1016/j.virol.2018.12.002.

15. Decaro, N.; Lanave, G.; Lucente, M.S.; Mari, V.; Varello, K.; Losurdo, M.; Larocca, V.; Bozzetta, E.; Cavaliere, N.; Martella, V.; et al. Mucosal Disease-Like Syndrome in a Calf Persistently Infected by Hobi-Like Pestivirus. *J. Clin. Microbiol.* **2014**, *52*, 2946–2954, doi:10.1128/JCM.00986-14.

16. Decaro, N.; Mari, V.; Pinto, P.; Lucente, M.S.; Sciarretta, R.; Cirone, F.; Colaianni, M.L.; Elia, G.; Thiel, H.-J.; Buonavoglia, C. Hobi-like Pestivirus: Both Biotypes Isolated from a Diseased Animal. *J. Gen. Virol.* **2012**, *93*, 1976–1983, doi:10.1099/vir.0.044552-0.

17. Mosena, A.C.S.; Falkenberg, S.M.; Ma, H.; Casas, E.; Dassanayake, R.P.; Booth, R.; De Mia, G.M.; Schweizer, M.; Canal, C.W.; Neill, J.D. Use of Multivariate Analysis to Evaluate Antigenic Relationships between US BVDV Vaccine Strains and Non-US Genetically Divergent Isolates. *J. Virol. Methods* **2022**, *299*, 114328, doi:10.1016/j.jviromet.2021.114328.

18. Neill, J.D.; Newcomer, B.W.; Marley, S.D.; Ridpath, J.F.; Givens, M.D. Genetic Change in the Open Reading Frame of Bovine Viral Diarrhea Virus Is Introduced More Rapidly during the Establishment of a Single Persistent Infection than from Multiple Acute Infections. *Virus Res.* **2011**, *158*, 140–145, doi:10.1016/j.virusres.2011.03.024.

19. RIDPATH, J.; NEILL, J.; VILCEK, S.; DUBOVI, E.; CARMAN, S. Multiple Outbreaks of Severe Acute BVDV in North America Occurring between 1993 and 1995 Linked to the Same BVDV2 Strain. *Vet. Microbiol.* **2006**, *114*, 196–204, doi:10.1016/j.vetmic.2005.11.059.

20. Meyer, C.; von Freyburg, M.; Elbers, K.; Meyers, G. Recovery of Virulent and RNase-Negative Attenuated Type 2 Bovine Viral Diarrhea Viruses from Infectious CDNA Clones. *J. Virol.* **2002**, *76*, 8494–8503, doi:10.1128/JVI.76.16.8494-8503.2002.

21. Moerlooze, L.D.; Lecomte, C.; Brown-Shimmer, S.; Schmetz, D.; Guiot, C.; Vandenbergh, D.; Allaer, D.; Rossius, M.; Chappuis, G.; Dina, D.; et al. Nucleotide Sequence of the Bovine Viral Diarrhoea Virus Osloss Strain: Comparison with Related Viruses and Identification of Specific DNA Probes in the 5′ Untranslated Region. *J. Gen. Virol.* **1993**, *74*, 1433–1438, doi:10.1099/0022-1317-74-7-1433.

22. Tzeng, H.-Y.; Chiou, Y.-Y.; Lin, F.-Y.; Yamada, Y.; Tsai, R.-S.; Ho, J.-J.; Tseng, C.-Y.; Lai, M.-C.; Cheng, Y.-H.; Chiou, H.-Y.; et al. Genomic Characterization and Evolutionary Analysis of Three Bovine Viral Diarrhea Virus 1b Strains from Taiwan, Highlighting Genomic Evidence of Cytopathic Evolution. *BMC Vet. Res.* **2025**, *21*, 680, doi:10.1186/s12917-025-05130-y.
